# Supraventricular Tachycardia: An Atypical Presentation of Thyroid Storm

**DOI:** 10.7759/cureus.25449

**Published:** 2022-05-29

**Authors:** Christopher P Austin, Mihir Odak, Steven Douedi, Swapnil V Patel

**Affiliations:** 1 Internal Medicine, Jersey Shore University Medical Center, Neptune, USA

**Keywords:** covid-19, thyroid storm, post sars-cov-2 complication, burch-wartofsky point scale, supraventricular tachycardia

## Abstract

Thyroid storm (or thyrotoxic crisis) is commonly defined as a life-threatening condition caused by the exaggeration of the clinical manifestations of thyrotoxicosis. Supraventricular tachycardia (SVT) is an atypical precipitating symptom of thyrotoxicosis that clinicians should be aware of. An empirically derived scoring system known as the Burch-Wartofsky Point Scale (BWPS) has been used by clinicians since the early 1990s. The BWPS considers an array of precipitating factors and the severity of symptoms of multiple organ decompensation. In recent years, there has been an increasing correlation between SARS-CoV-2 and thyroid pathologies. We present a case of an unresponsive elderly male with a recent coronavirus disease 2019 (COVID-19) infection presenting with SVT and a BWPS score of 45, highly indicative of a thyroid storm.

## Introduction

Thyrotoxicosis is a serious complication of hyperthyroidism. Thyroid storm presents with non-specific clinical symptoms that can lead to fatal tachyarrhythmias if left untreated. Supraventricular tachycardia (SVT) is an uncommon presentation of thyroid storm, with an incidence of 2-20% [1]. SARS-CoV-2 has a reported association with thyroid abnormalities, particularly hyperthyroid states [2-8]. We present a case of an unresponsive elderly male with a recent coronavirus disease 2019 (COVID-19) infection presenting with SVT and a Burch-Wartofsky Point Scale (BWPS) score of 45, highly indicative of a thyroid storm.

## Case presentation

A 76-year-old male with a medical history significant for abdominal aortic aneurysm status post repair, cerebrovascular accident status post tracheostomy placement, and recent COVID-19 pneumonia status post monoclonal antibody infusion presented with tachycardia to the 150s and lethargy. His vital signs were notable for a heart rate of 155 beats per minute, blood pressure of 74/52 mmHg, temperature of 101.2°F, and saturation of 98% on 40% fraction of inspired oxygen (FiO2) and positive end-expiratory pressure (PEEP) of 5 via a ventilator.

**Figure 1:**
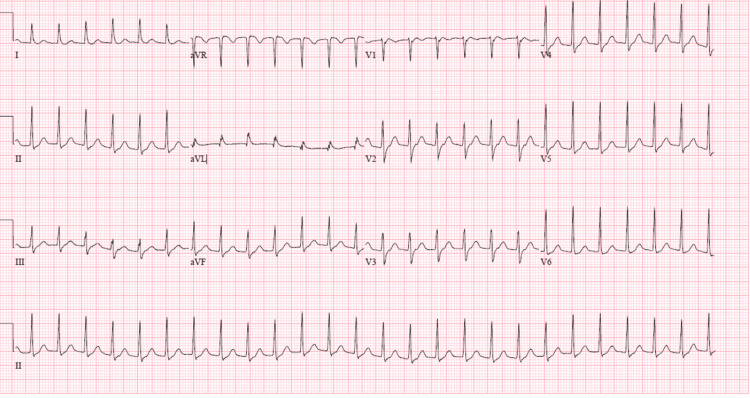
The patient's ECG at presentation was significant for supraventricular tachycardia but was otherwise normal.

Upon physical examination, he was unresponsive and ill-appearing, his mucous membranes were dry, a tracheostomy and percutaneous endoscopic gastrostomy were present, and he was tachycardic and mildly diaphoretic. The thyroid exam was deferred due to tracheostomy and collar placement. Labs at that time showed normal white blood cells, increased lactic acid, decreased thyroid-stimulating hormone, increased free thyroxine (T4), and normal total triiodothyronine (T3) (Table 1). Other labs were within normal limits. Blood cultures and urine cultures were negative. A portable X-ray of the chest showed no pneumothorax or congestive failure and no infiltrates or effusions. An electrocardiogram (ECG) was significant for SVT but was otherwise normal (Figure 1).

**Table 1:** The patient's laboratory findings at presentation.

Laboratory study	Results	References
White blood cells (10*3u/L)	8.3 (10*3u/L)	4.5-11.0 (10*3u/L)
Lactic acid (mmol/L)	2.3 (mmol/L)	0.5-2.0 (mmol/L)
Thyroid-stimulating hormone (uIU/mL)	<0.010 (uIU/mL)	0.300-4.500 (uIU/mL)
Free thyroxine (T4) (ng/dL)	1.50 (ng/dL)	0.50-1.26 (ng/dL)
Total triiodothyronine (T3) (ng/dL)	91 (ng/dL)	76-181 (ng/dL)

At this point, the patient was shocked. He had a score of 45 on the BWPS, which was highly suggestive of a thyroid storm. An endocrine consultation was placed, which deemed the patient to likely have thyrotoxicosis and currently in thyroid storm with unknown etiology. The patient was promptly started on propylthiouracil, hydrocortisone, cholestyramine, and propranolol. Thyroid ultrasound was not done due to tracheostomy and neck collar. Labs were found to be within normal limits for thyroid-stimulating immunoglobulin and thyroid receptor antibodies. Thyroid function tests were trended over the next four days, which saw a subsequent reduction in T4 and T3. At this point, the patient was afebrile with normal sinus rhythm and was taken off of propylthiouracil and cholestyramine and switched to methimazole. The patient was ultimately discharged at this point in stable condition back to rehab.

## Discussion

Thyroid storm (or thyrotoxic crisis) is defined as a life-threatening condition caused by the exaggeration of the clinical manifestations of thyrotoxicosis [9]. Commonly presenting as sinus tachycardia or atrial fibrillation in 10-25% of patients, SVT is an atypical yet life-threatening precipitating symptom of thyroid storm [1]. The diagnosis of thyroid storm can be difficult in the acute setting, with pending thyroid function tests, due to the nonspecific nature of symptoms seen. An empirically derived scoring system known as the BWPS has been used by clinicians since the early 1990s. The BWPS considers an array of precipitating factors and the severity of symptoms of multiple organ decompensation. These factors are composed of thermoregulatory dysfunction, tachycardia, atrial fibrillation, disturbances of consciousness, congestive heart failure, and gastro-hepatic dysfunction [10]. Our patient in this case report received a BWPS score of 45 due to cardiovascular tachycardia ≥ 140 (+25) and a temperature of 101.2°F (+20). Treatment of thyroid storm is divided into several categories: inhibiting new thyroid hormone synthesis, inhibiting thyroid hormone release, inhibiting the peripheral effect of thyroid hormone, inhibiting enterohepatic circulation of thyroid hormone, and, in refractory cases, therapeutic plasma exchange can be used [11]. With associated in-hospital mortality of 10.1%, prompt initiation of treatment is imperative [12]. Our patient was promptly started on a standard regimen for treating thyroid storm consisting of propylthiouracil, hydrocortisone, cholestyramine, and propranolol. This regiment worked with great success and thyroid function returned to normal ranges by day four.

There has been an increasing correlation between COVID-19 and thyroid pathologies [2-8]. It has been well established that COVID-19 uses angiotensin-converting enzyme 2 (ACE2) combined with the transmembrane protease serine 2 (TMPRSS2) as the key molecular complex to enter and infect the host cells [13]. Both ACE2 and TMPRSS2 expression levels are high in the thyroid gland with thyroid ACE2 expression levels being positively and negatively linked to immune signatures in males and females, respectively, thus contributing to explaining different immune responses and the resultant distinct thyroid manifestations [13]. Our patient was found to be positive for COVID-19 four weeks prior to presentation and had no pre-existing thyroid pathologies. Both thyroid-stimulating immunoglobulin (<89; reference range < 140% baseline) and thyroid receptor antibody (<1.00; reference range ≤ 2.00 IU/L) were normal, making Hashimoto’s and Graves' disease unlikely. Unfortunately, the patient was unresponsive, noted to be awake, alert, and oriented (AAO) x 0 secondary to a cerebrovascular accident two months prior status post tracheostomy, and with a neck collar. Therefore, the workup for thyroid pathology was limited. Patients presenting with post-viral infection and thyrotoxicosis often have subacute granulomatous thyroiditis that presents with a tender thyroid gland, which we were unable to assess properly in this patient. However, there have been a few reported cases of thyroid storm in patients following infection from COVID-19. In our patient, it is believed that he went into thyroid storm status post COVID-19 infection.

## Conclusions

SVT is an atypical precipitating symptom of thyrotoxicosis that clinicians should be aware of. Any new onset SVT should be followed up with a calculation of the BWPS. The BWPS is composed of nonspecific symptoms that could be attributed to other etiologies, but if it is elevated above 45, treatment should be started for a thyroid storm. There should be an increased suspicion of a hyperthyroid state in patients with recent COVID-19 infection, as these states have become increasingly more common. Our patient had no known hyperthyroidism until being worked up after presenting with SVT, fevers of unknown origin, and a calculated BWPS score of 45. Our patient was successfully treated immediately and discharged in stable condition just four days later.
